# Establishment of targeted mutagenesis in soybean protoplasts using CRISPR/Cas9 RNP delivery via electro−transfection

**DOI:** 10.3389/fpls.2023.1255819

**Published:** 2023-09-29

**Authors:** Saminathan Subburaj, Sarah Zanon Agapito-Tenfen

**Affiliations:** NORCE Norwegian Research Centre AS, Climate & Environment Department, Siva Innovasjonssenter, Tromsø, Norway

**Keywords:** CRISPR/Cas9 RNPs, gene-editing, neon electroporation system, soybean, protoplasts, target-deep sequencing, trait improvement

## Abstract

The soybean (*Glycine max* L.) is an important crop with high agronomic value. The improvement of agronomic traits through gene editing techniques has broad application prospects in soybean. The polyethylene glycol (PEG)-mediated cell transfection has been successfully used to deliver the CRISPR/Cas9-based ribonucleoprotein (RNP) into soybean protoplasts. However, several downstream analyses or further cell regeneration protocols might be hampered by PEG contamination within the samples. Here in this study, we attempted to transfect CRISPR/Cas9 RNPs into trifoliate leaf-derived soybean protoplasts using Neon electroporation to overcome the need for PEG transfection for the first time. We investigated different electroporation parameters including pulsing voltage (V), strength and duration of pulses regarding protoplast morphology, viability, and delivery of CRISPR/Cas9. Electroporation at various pulsing voltages with 3 pulses and 10 ms per pulse was found optimal for protoplast electro-transfection. Following electro-transfection at various pulsing voltages (500 V, 700 V, 1,000 V, and 1,300 V), intact protoplasts were observed at all treatments. However, the relative frequency of cell viability and initial cell divisions decreased with increasing voltages. Confocal laser scanning microscopy (CLSM) confirmed that the green fluorescent protein (GFP)-tagged Cas9 was successfully internalized into the protoplasts. Targeted deep sequencing results revealed that on-target insertion/deletion (InDel) frequencies were increased with increasing voltages in protoplasts electro-transfected with CRISPR/Cas9 RNPs targeting *constitutive pathogen response 5* (*CPR5*). InDel patterns ranged from +1 bp to −6 bp at three different target sites in *CPR5* locus with frequencies ranging from 3.8% to 8.1% following electro-transfection at 1,300 V and 2.1% to 3.8% for 700 V and 1,000 V, respectively. Taken together, our results demonstrate that the CRISPR/Cas9 RNP system can be delivered into soybean protoplasts by the Neon electroporation system for efficient and effective gene editing. The electro-transfection system developed in this study would also further facilitate and serve as an alternative delivery method for DNA-free genome editing of soybean and other related species for genetic screens and potential trait improvement.

## Introduction

The CRISPR/Cas9 system has become a versatile technology in plant breeding and functional genomics due to its design simplicity and high efficiency in genome editing (Jiang and Doudna, 2017; Liu et al., 2022). Genome editing via CRISPR/Cas9 system uses single guide RNA (sgRNA) and Cas9 protein to make mutation events including insertions and deletions (InDels) at desired locations in the host genome through non-homologous end-joining (NHEJ) or homology-directed repair (HDR) pathways (Jinek et al., 2013). The CRISPR/Cas9 system enables targeted modifications in the genome by gene knock-in/out, base editing, prime editing, and guided transcriptional regulation (Feng et al., 2013; Zong et al., 2017; Mao et al., 2018; Lin et al., 2020; Rezazade Bazaz and Dehghani, 2022). Therefore, an optimized CRISPR/Cas9 cell system would facilitate efficient genetic screens for target genes and off-target activity in the plant genomes, thereby accelerating breeding and biosafety research.

The DNA expression cassettes of the CRISPR/Cas9 system can be transformed into plant cells using the most common delivery methods including *Agrobacterium*-mediated (Zhang et al., 2019), virus-mediated (Lei et al., 2022), particle bombardment (Hamada et al., 2018), and polyethylene glycol (PEG)-mediated transfection (Wu et al., 2020). Despite the fact that *Agrobacterium* and biolistic methods have been widely used in many organisms to deliver components for gene editing (Sandhya et al., 2020; Ghogare et al., 2021), their inadvertent incorporation of transposable elements (T-DNA) into the host genomes results in continued expression of CRISPR machinery, often resulting in unintended off-target mutation and genomic rearrangement (Zhang et al., 2018; Jupe et al., 2019; Chu and Agapito-Tenfen, 2022). Although virus-induced gene editing systems have many advantages including high editing efficiency and without integration of exogenous DNA into the host genome, their capacity to deliver the entire CRISPR/Cas9 system into plant cells is lower, which limits their application as a delivery method (Liu and Zhang, 2020; Zhang et al., 2022). Developing gene-edited lines without CRISPR T-DNA remnants in the host genome is gaining importance in the global genetically modified (GM) regulatory landscapes. Therefore, the DNA-free Cas9 ribonucleoprotein (RNP) (gRNA precomplexed to Cas9 nuclease) delivery into protoplasts has been adopted as a versatile method for genome editing of a diverse range of plants.

The direct delivery of RNPs into plant cells can be achieved by PEG-mediated as well as electroporation-mediated transfection or electro-transfection. PEG-mediated transfection has been widely used to deliver RNPs into plant protoplasts including *Arabidopsis*, tobacco and rice (Woo et al., 2015), petunia (Subburaj et al., 2016), apple and grape (Malnoy et al., 2016), wheat (Liang et al., 2017), cabbage (Murovec et al., 2018), pepper (Kim et al., 2020), maize (Sant’Ana et al., 2020), tomato (Nicolia et al., 2021), and soybean (Subburaj et al., 2022). PEG might exhibit incompatibility to several downstream analyses (e.g., proteomic analysis) and may cause some degree of cell cytotoxicity, with toxic effects occasionally seen in protoplasts when transfection with PEG is performed (Tyagi et al., 1989; Masani et al., 2014). Electro-transfection is another direct delivery method for the efficient transfection of the CRISPR/Cas9 system to living cells, and it has been widely used in the transformation of human and mouse primary T cells (Rupp et al., 2017; Seki and Rutz, 2018). Electro-transfection of RNPs has been demonstrated successfully in the microalga model *Chlamydomonas reinhardtii* (Baek et al., 2016; Shin et al., 2016). In addition, electroporation was also used for the delivery of RNPs (cabbage protoplast) and plasmid DNA containing CRISPR reagents (oil palm protoplast and wheat microspores) to plant cells (Bhowmik et al., 2018; Lee et al., 2020; Yeap W. et al., 2021). In soybean protoplasts, the transient expression of electroporated DNA has been reported by several studies (Dhir et al., 1991; Christou et al., 1987; Lin et al., 1987), but these available methods have not been adopted and or updated for targeted genome editing through transient introduction of RNA-guided endonuclease (RGEN) RNPs into soybean protoplasts.

Soybean is an economically important agronomic crop with high protein and oil, and several genetic engineering approaches have been made to improve the soybean traits (Rahman et al., 2023). Very recently, an efficient DNA-free genome editing platform for soybean protoplast systems using direct delivery of Cas9-RNP through PEG-mediated transfection was established by our research group (Subburaj et al., 2022). To date, no standardized protocols exist to transfect RNPs to soybean protoplasts through electroporation with reasonable mutation efficiency, which would greatly facilitate the CRISPR/Cas9 system to soybean protoplasts of different genetic backgrounds and further downstream analysis such as the impact of CRISPR exposure to soybean proteome.

In the present study, we report the establishment of a CRISPR/Cas9 RNP delivery system that facilitates efficient RNA-based genome editing in soybean protoplasts using electro−transfection. Using the Neon electroporation system (Thermo Fisher Scientific, Waltham, MA, USA), we investigated the important electrical factors including voltage strength and pulse duration on protoplasts, and we analyzed protoplasts of post-electroporation. With the established electrical parameters, we successfully demonstrated the delivery of exogenous green fluorescent protein (GFP)-Cas9 into the nucleus of soybean protoplasts using electro-transfection. By targeting the *Glycine max CPR5* locus (*GmCPR5*) that is associated with soybean trichome growth, we validated the mutations at three different sgRNA targeted sites and determined the mutagenesis efficiency of CRISPR/Cas9 in soybean protoplasts by targeted deep sequencing.

## Materials and methods

### Plant material and protoplast extraction

The soybean (*G. max*) seeds cv. OAC Bayfield was planted and grown in soil pots under a photoperiod of 8-h light and 16-h dark at 25°C in a growth chamber (Enviro Plant®) for 3 weeks. The newly expanded trifoliate leaves from 14–16-day- old soybean seedlings were used for protoplast isolation. The extraction of protoplasts was carried out according to our previous study (Subburaj et al., 2022) with minor modifications. Briefly, 12–18 trifoliate leaves were sliced into 0.2–0.4- mM strips and were agitated in 20 ml of VCP (Viscozyme® L, Celluclast® 1.5L, and Pectinex® Μltra SPL) (Sigma-Aldrich, Darmstadt, Germany) cell wall digesting enzyme solution for 4–5 h at 25°C in the dark to isolate protoplasts. After digestion, the solution was filtered through 0.75- µm nylon mesh, and the filtrate was pelleted at 600 rpm for 5 min. Harvested protoplasts were rinsed thrice with 10 ml of 9M cell and protoplast washing (CPW) solution, followed by resuspending and centrifugation. The washed and purified protoplasts were kept in ice for 1 h prior to further use.

### Target gene and guide RNA selection

In this study, we chose *GmCPR5* as the target gene, as it was already attempted to make site-directed mutations in their coding region using CRISPR/Cas9 RNP through the PEG-mediated delivery method; to accomplish this, we designed five candidate gRNAs (denoted as T1–T5) against *CPR5* coding region in our previous study (Subburaj et al., 2022). In the present study, we selected and used best gRNAs of T1 (5′-AGGCTGCGGCGTTCAAACGACGG-3′), T3 (5′-GTCTCCCAGTCATCTTTCGATGG-3′), and T5 (5′-AGCTTTAGTAATCCGCTCGTAGG-3′) due to their higher mutation frequency at the *CPR5* locus (Subburaj et al., 2022). We carried out the *in vitro* transcript synthesis and purification of these gRNAs as reported previously (Subburaj et al., 2022).

### Electroporation-mediated soybean protoplast transformation

The purified protoplasts were centrifuged and resuspended in 9M CPW solution and then counted using a hemocytometer. CPW solution with a volume of 100 µl containing approximately 4 × 10^5^ protoplasts was transferred to a 1.5-ml Eppendorf tube as needed for each transfection. After brief centrifugation, CPW solution was removed, and protoplasts were resuspended in 80 µl of transfection buffer, which included R buffer (Neon suspension buffer), MMG solution (4 mM of MES, 0.4 M of mannitol, and 15 mM of MgCl), and HEPES electroporation buffer (10 mM of Hepes (pH 7.2), 0.2 M of mannitol, 5 mm of CaCl_2_, and 150 mM of NaCl). Next, 20 µl of RNP complex was added to 80 µl of protoplast resuspension to bring a final volume of 100 µl. Then, the electroporation was carried out using the Neon™ Transfection System (Invitrogen, Carlsbad, CA, USA) following the manufacturer’s instructions in 100-µl volumes. Protoplasts were electroporated with various voltages (500 V, 700 V, 1,000 V, and 1,300 V), pulse (1 and 3), and width (10 ms, 20 ms, and 30 ms) to optimize and obtain efficient transfection conditions as described in the Results section. With the optimized electro-transfection conditions, 20 µl of RNP complexes (1:3 molar ratio) consisted of 10 µg of ready-to-use GFP-tagged Cas9 from Sigma-Aldrich (CAS9GFPPRO), and 30 µg of sgRNA was electrophoretically introduced into 80 µl of resuspended protoplasts. Control and electroporated cells were immediately transferred in a 12-well poly-d-lysine-coated cell plate with 0.5 ml of precooled KP8 liquid medium (Kao, 1977) (supplemented with 9% mannitol and 3% sucrose). Then, the plates were kept on ice for 1–2 h and shifted to 25°C in darkness for 16–24 h prior to DNA extraction. Control and transfected cells were further cultured in 1 ml of KP8 liquid medium for 4 weeks at 25°C in the dark.

### Microscopic observation of protoplasts

The bright-field images of isolated and transfected cells were analyzed using Motic AE2000 inverted microscope and captured with Moticam (Motic Hong Kong Limited, Hong Kong). The viability of control and electro-transfected cells was assessed by 0.5% fluorescein diacetate (FDA) staining, observed under reflected light with Axio Vert.A1 (FL-LED Stand) inverted light microscope, and captured via Axiocam 202 mono (Carl Zeiss MicroImaging GmbH, Oberkochen, Germany). The fluorescence images of GFP-tagged Cas9 were acquired under bright field and electronically switchable illumination and detection module (ESID) using confocal laser scanning microscopy (CLSM) (LSM 800; Carl Zeiss) using a diode laser (488-nm line) with a 40× objective lens. The viability and transfection efficiency were calculated as the number of fluorescent protoplasts divided by the total number of protoplasts in one representative microscope field (Adedeji et al., 2020; Subburaj et al., 2022).

### T7E1 validation and targeted deep sequencing

The genomic DNA from control and electro-transfected samples was prepared using Plant DNAzol™ Reagent (Invitrogen Co., Carlsbad, CA, USA). For each sgRNA (T1, T3, and T5), the target-specific nested PCR primers were designed in *GmCPR5* loci (**Supplementary Table 1**), and the extracted DNA was PCR amplified using designed primers. Targeted mutagenesis in PCR products of both control and electro-transfected protoplasts was detected by T7 endonuclease I (T7E1) assay. Further, the InDels at the targeted locations were also analyzed by targeted deep sequencing using the Illumina NovaSeq™ 6000 platform at Novogene Europe (Cambridge Science Park, UK) as described previously (Subburaj et al., 2022). CRISPR/Cas9 RNP induced InDels at *GmCPR5* loci for each sgRNA were determined using Cas-Analyzer from the CRISPR RGEN tools (http://www.rgenome.net/cas-analyzer/) (Subburaj et al., 2016; Subburaj et al., 2022). Briefly, the raw data FASTQ files along with basic information about query sequences were submitted in the Cas-Analyzer software with the following analysis parameters: comparison range of 40 and minimum frequency of 1. Following submission, the total number of reads, the number of reads with InDels, and the calculated InDel frequency (defined as the percentage by dividing the count of reads that contained InDels at the target site by the total number of reads) were obtained from the output of a summarized result table.

## Results

### Isolation of protoplasts from trifoliate leaves

**Figure 1:**
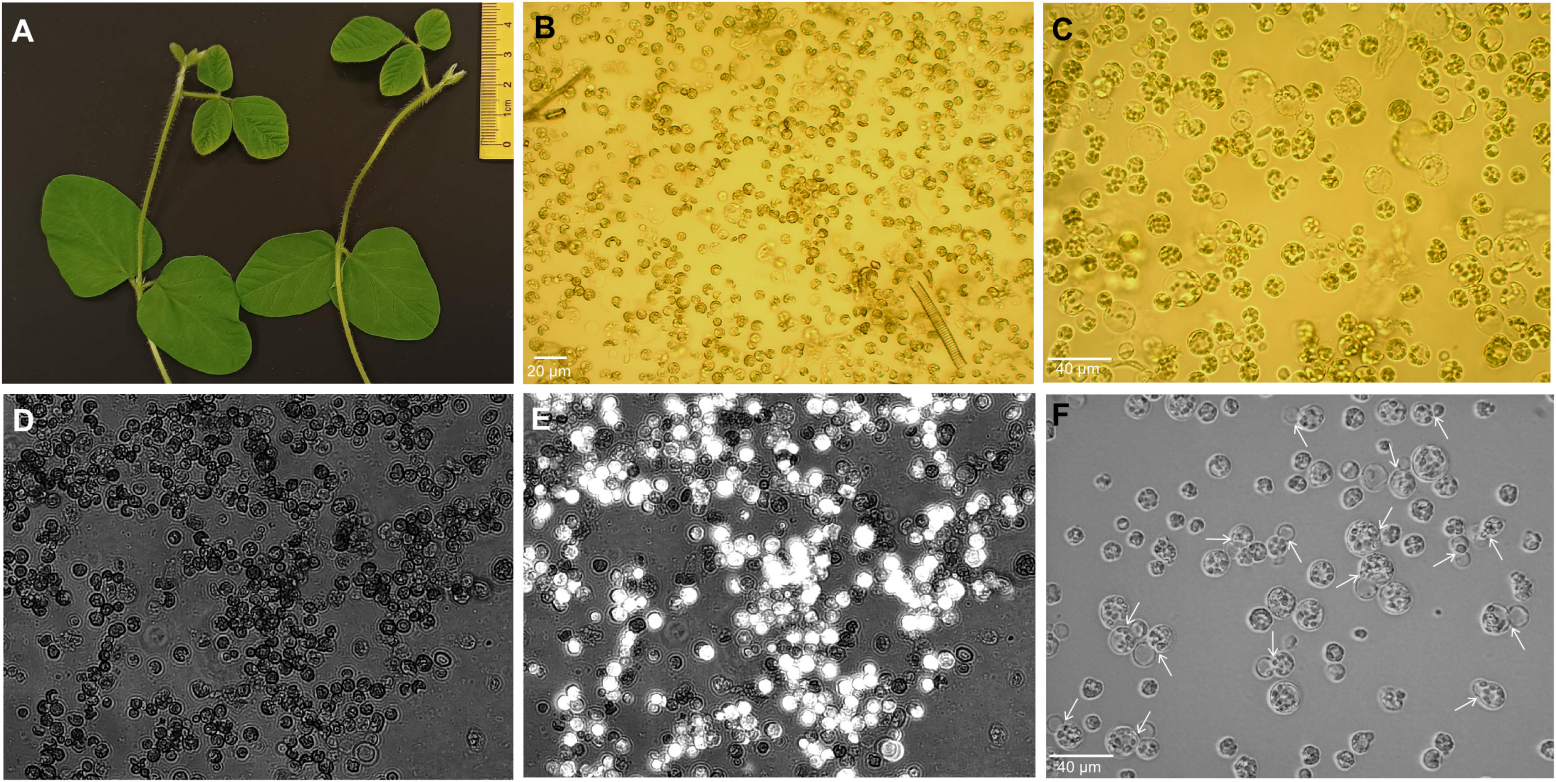
Isolation of protoplasts from trifoliate leaves of soybean plantlets. **(A)** Fifteen-day-old plants showing trifoliate leaves of suitable size. **(B, C)** Protoplasts of freshly extracted **(B)** and purified cells **(C)** under the Motic AE2000 inverted microscope with × 20 and × 40 objectives, respectively. Black scale bar, 30 µm. **(D, E)** The protoplast viability was assessed by FDA staining and observed under both bright field **(D)** and fluorescence channel, and simultaneously merged images are depicted **(E)** using Axio Vert.A1 inverted microscope with a × 20 objective. **(F)** Division of protoplasts (shown by white arrows) at 4 days after isolation in culture medium. FDA, fluorescein diacetate.

The initial electroporation experiments showed that trifoliate protoplasts are the most suitable for electro-transfection compared to unifoliate cells, as they were severely damaged after electroporation. In this study, we efficiently isolated the protoplast from 15- day-old trifoliate leaves with a duration of 5–6 h of enzyme digestion (**Figures 1A, B**). The yield of protoplasts was approximately 2 × 10^6^ cells per gram fresh weight. Isolated protoplasts were 10 μm to 50 μm in size, and most were spherical in shape (**Figure 1C**). Following FDA staining, it was determined that 70% ± 2.1% of the protoplasts were alive (**Figures 1D, E**). The isolated protoplasts were cultured in KM medium, and first cell divisions were noted after 4–6 days of culture initiation (**Figure 1F**), indicating that the trifoliate leaf-derived protoplasts could be suitable for the electroporation-mediated transformation.

### Electro-transfection of protoplasts using Neon electroporation system

**Figure 2:**
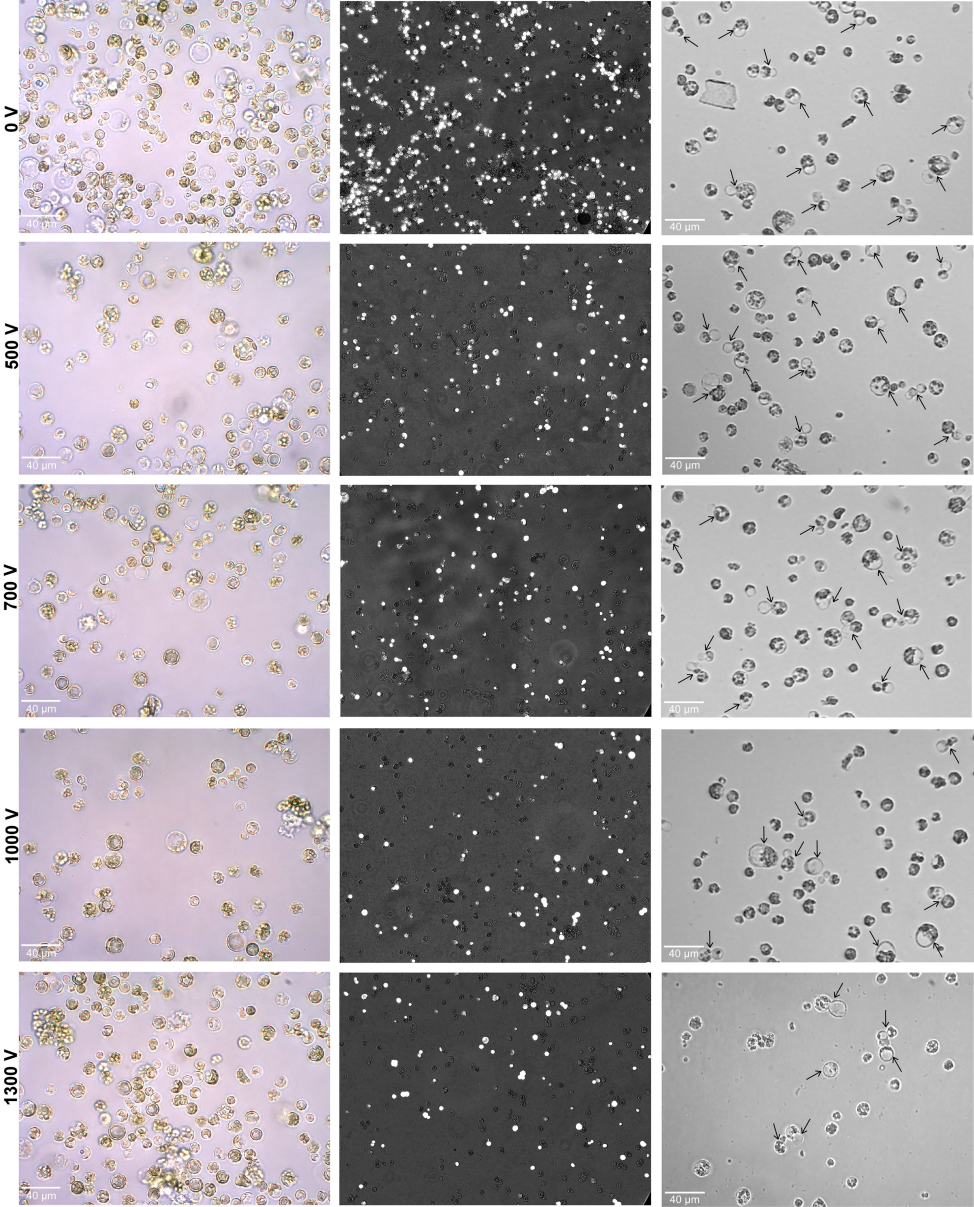
Effect of various pulsing voltage on the protoplasts morphology, viability, and cell division efficiency following electro-transfection. Left panel: representative images of electroporated protoplasts after 24 h are shown. Middle panel: the merged fluorescence images (under bright and fluorescence field using Axio Vert.A1 inverted microscope with a × 20 objective) showing the FDA-stained viable cells after 24 h of electroporation. Right panel: images showing primary divisions (shown by black arrows) of electroporated protoplasts at 4 days after culture initiation. FDA, fluorescein diacetate.

To establish an electroporation-mediated soybean protoplast transformation using the Neon system, the electro-transfection method was optimized based on cabbage and wheat protocols where transfection efficiency reaches 3.4% (1 pulse of 1,000 V, 20 seconds each) and 2.2% (3 pulses of 500 V, 30 seconds each), respectively (Bhowmik et al., 2018; Lee et al., 2020). Following cabbage and wheat electroporation conditions, initial soybean testing failed, as we could not detect or could only detect a few GFP fluorescent spots in electroporated cells. Subsequently, the various pulse strengths (1–3) along with voltages (500–1,300 V) and time duration (10–30 ms) were optimized. It was found that 3 pulses of 500 V and 10 ms per pulse were sufficient to transport the exogenous GFP-Cas9 with lower cell death rates. The effects of various ranges of pulsing voltages were further evaluated with optimized 3 pluses and 10 ms per pulse in the protoplasts (100 μl of 4 × 10^5^ cells) for 500 V, 700 V, 1,000 V, and 1,300 V. As shown in **Figure 2**, the protoplasts’ morphology, viability, and cell division efficiency were examined after electroporation. It was noted that intact protoplasts with a large and round shape were observed at all the applied voltages. However, at 1,000 and 1,300 V, a certain proportion of broken and debris of dead cells was also noted.

The calculated relative frequency of cell viability was 86.5% ± 2.4% under 0 V compared to the non-treatment control of 100%. Under various voltage treatments, the viability of cells was decreased with the increase in pulsing voltage. The observed relative frequency of viable rates for treatments under 500 V, 700 V, 1,000 V, and 1,300 V were 76.7% ± 2.8%, 66% ± 1.4%, 59.1% ± 1.2%, and 57.6% ± 1.1%, respectively (**Figure 2**). Upon culturing of electroporated protoplasts, the primary cell divisions were noted in all treatments. However, a higher proportion of cell divisions was only observed at 0 V and 500 V when compared to the higher voltages of 700 V, 1,000 V, and 1,300 V (**Figure 2**).

**Figure 3:**
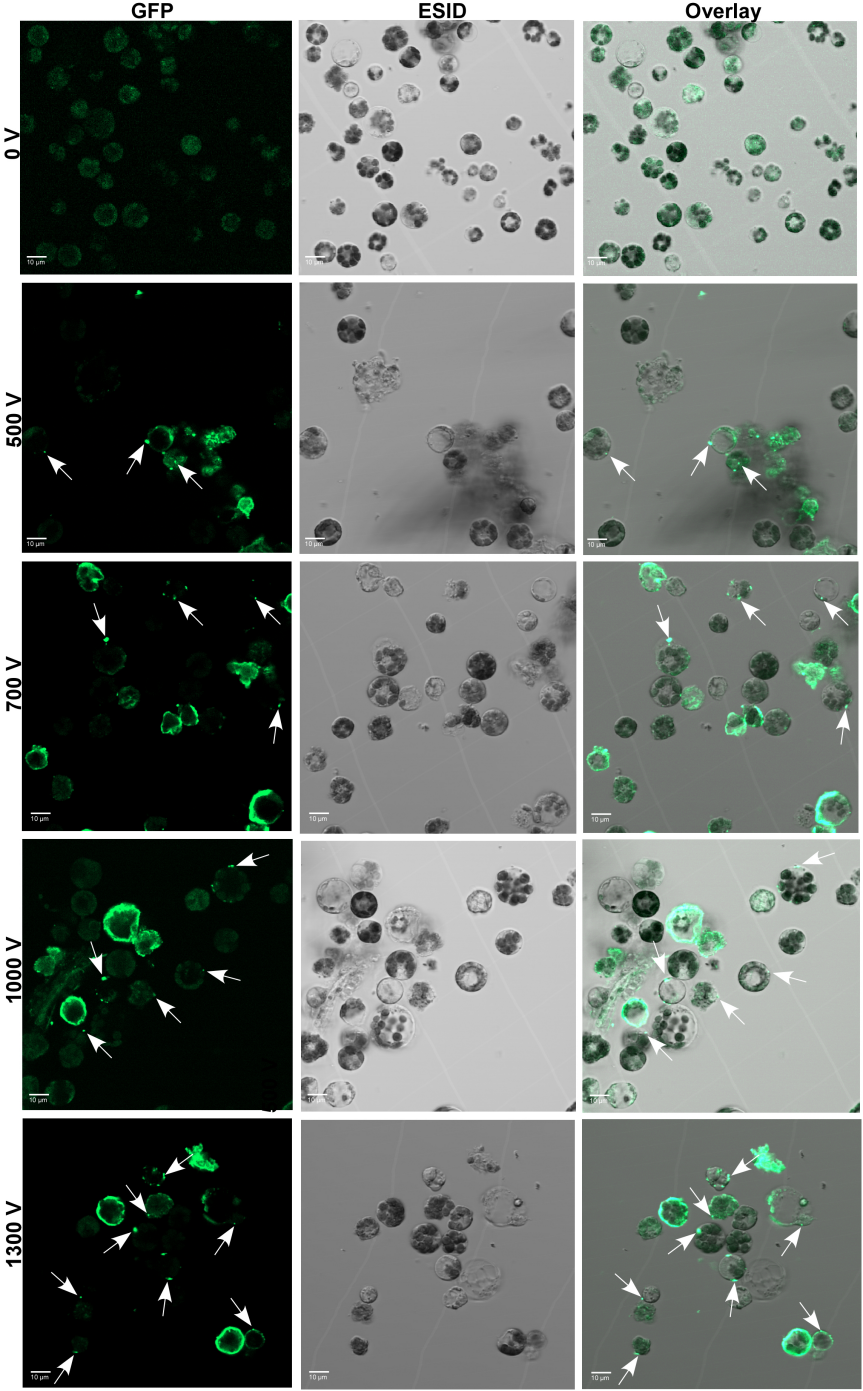
Delivery and cellular localization of Cas9-GFP to soybean protoplasts through electro-transfection. GFP-Cas9 in electro-transfected (0 V to 1,300 V) protoplasts at 24 h after electroporation was seen using a laser scanning confocal microscope under GFP (left panel) and bright field of ESID channel (middle panel). Right panel: representative overlay images of GFP and bright field are shown. White arrows show the location of internalized GFP-Cas9. GFP, green fluorescent protein; ESID, electronically switchable illumination and detection module.

To further optimize and validate the delivery of Cas9 protein, a fixed amount of 10 µg of GFP-conjugated Cas9 was electro-transfected into protoplasts with the abovementioned pulsing voltages. After 24 h of electroporation, the internalization of GFP-tagged Cas9 inside the protoplasts was confirmed under the CLSM microscope (**Figure 3**). Regardless of voltage, the GFP-Cas9 was successfully localized inside of cells. However, the calculated internalization efficiency of GFP-Cas9 with an unsupervised eye was ⪸ 40% under 1,300 V compared to other treatments such as 500 V, 700 V, and 1,000 V where there was only ∼ 20%–23% noted. These results suggest that the established soybean protoplast transfection using the Neon electroporation system can be suitable for the delivery of RNPs into protoplasts.

### RNP-based targeted mutagenesis of *CPR5* in soybean via electro-transfection

**Figure 4:**
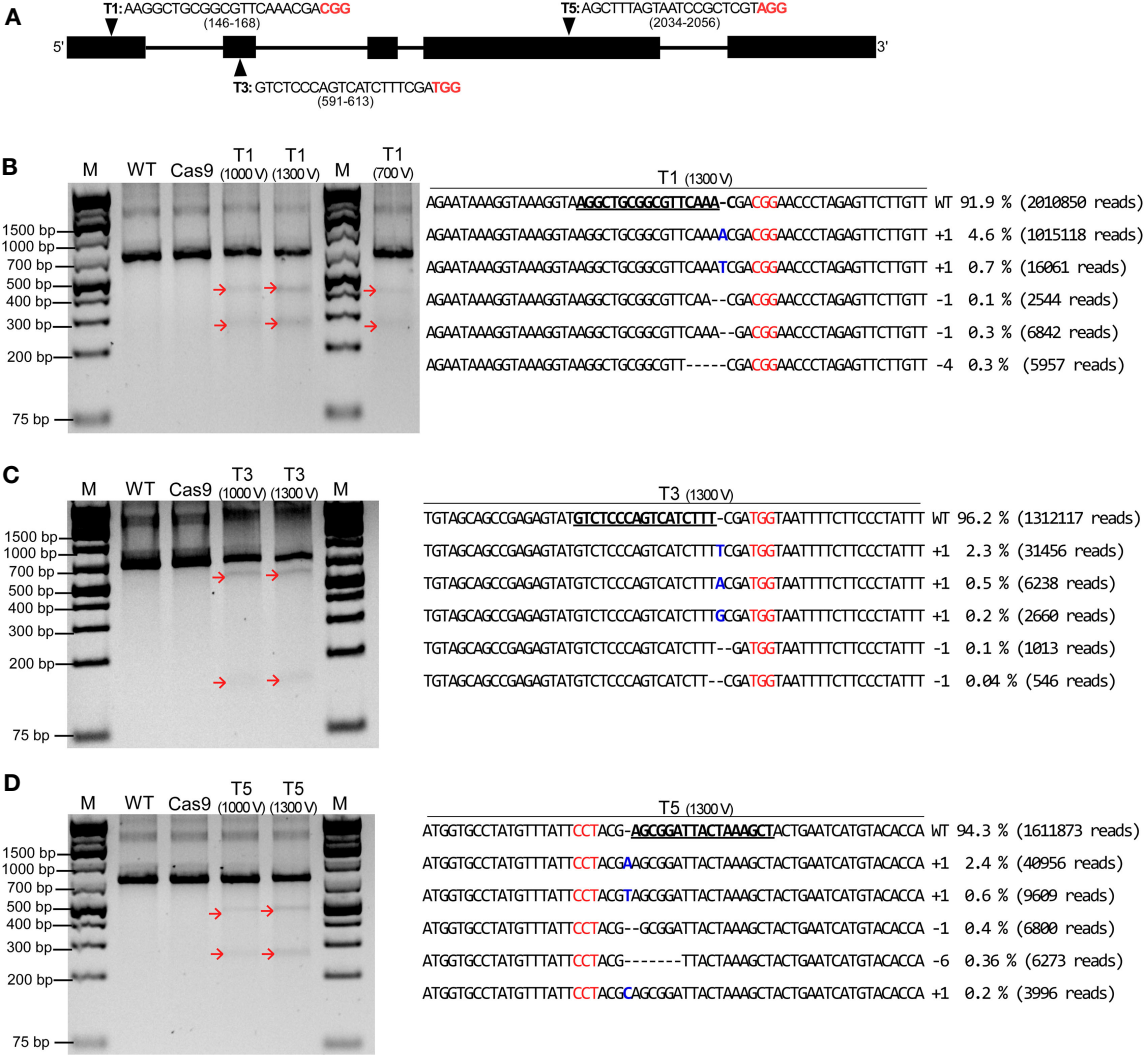
CRISPR/Cas9-mediated editing of the exogenous *GmCPR5* gene in soybean protoplasts using electro-transfection. **(A)** *GmCPR5* locus, location of target sites (T1, T3, and T5), and their gRNA sequences. **(B–D)** Results of T7E1 endonuclease assay for target sites T1, T3 and T5. Lane M: a DNA ladder. Lane WT: non- transfected wild type (control). Lane Cas9: transfected with SpCas9 only. Lanes T1–T5: electro-transfected with RNPs at 700 V, 1,000 V, and 1,300 V. Red arrows indicate the T7E1-mediated cleaved bands. The mutation patterns observed by targeted deep sequencing for the corresponding target sites of T1–T5 at *GmCPR5* loci by electro-transfection at 1,300 V are shown on the right panel. Wild-type (WT) nuclease target sequences are in bold and underlined. PAM sites are denoted in red. RNPs, ribonucleoproteins; gRNA, guide RNA.

To demonstrate whether the established electro-transfection system can be employed for RGEN RNP-mediated genome editing in soybean, three sgRNAs were chosen and prepared based on our previous study (Subburaj et al., 2022), namely, T1, T3, and T5 to target exons 1, 2, and 4 of *CPR5* gene, respectively (**Figure 4A**). RNP complex consisting of a 1:3 molar ratio of Cas9 (10 µg) and synthesized sgRNAs (30 µg) were electro-transfected into protoplasts using the Neon system at different pulsing voltages as mentioned earlier. Following transfection, the genomic DNA was extracted from control and transfected samples after 24 h of incubation. The targeted sites were PCR amplified using designed nested primers (**Supplementary Table 1**), and a T7E1 assay was carried out for preliminary detection of RNP-induced mutations for electro-transfected samples under different pulsing voltages. Upon agarose gel electrophoresis of T7E1-digested PCR products, cleaved PCR fragments at expected sizes were noted for all the RNP electro-transfected samples under 1,000 V and 1,300 V (**Figures 4B–D**). In addition, there were no cleaved PCR products observed for all the target sites under 500 V and or 700 V, except for T1 under 700 V; the same was observed for the wild type and the Cas9 alone transfected samples. This suggests that RNPs successfully induced site-specific double-strand breaks followed by DNA repair mechanisms within the *GmCPR5* locus in soybean.

To estimate InDel frequency and characterization of mutation patterns, targeted deep sequencing was performed on the DNA samples with positive results from the T7E1 assay. The raw data from targeted deep sequencing were submitted to the National Center for Biotechnology Information (NCBI) Bioproject (http://www.ncbi.nlm.nih.gov/bioproject/) under the accession number PRJNA983990. Targeted deep sequencing results confirmed that the InDels present at all three target sites including T1, T3, and T5 under different pulsing voltages, similar to that in the T7E1 cleavage assay. As shown in **Table 1**, the percentage of InDel (mutation) frequency of each target site was estimated by dividing the number of mutated InDel sequences by the number of total sequences in the *CPR5* target sequences using the Cas-Analyzer tool. The target sites of T1, T3, and T5 have InDel frequencies that ranged from a minimum of 2.1% to a maximum of 8.1% with an average frequency of 4.21% ± 0.73% in the *GmCPR5* locus. It was found that the estimated InDel frequencies were increased with the increase in pulsing voltage. For the T1 target site, InDel frequency following electro-transfection at 700 V, 1,000 V, and 1,300 V were 2.1%, 3.7%, and 8.1%, respectively. Similarly, in T3 and T5, the frequency was increased from 2.1% and 3.8% (at 1,000 V) to 3.8% and 5.7% at 1,300 V, respectively (**Table 1**).

**Table 1:** Estimation of mutation rates at *GmCPR5* locus following electro-transfection of Soybean protoplasts.

Protoplast samples	Wild-type negative control				
	Total	InDel	InDel frequency (%)	Insertion^a^	Deletion^b^
T1 (0 V)	1,713,502	164	0.009	5	159
T3 (0 V)	1,207,415	82	0.006	3	79
T5 (0 V)	1,285,534	239	0.018	0	239
Average^c^	1,402,150 ± 128,435.4	161 ± 37.01	0.011 ± 0.003	2.66 ± 1.18	159 ± 37.71
Cas9 RNP transformants					
T1 (700 V)	2,233,489	47,431	2.1	47,238	193
T1 (1,000 V)	1,964,762	72,550	3.7	39,940	32,610
T1 (1,300 V)	2,186,990	176,140	8.1	142,852	33,288
T3 (1,000 V)	736,342	16,884	2.3	14,417	2,467
T3 (1,300 V)	1,363,363	51,246	3.8	47,636	3,610
T5 (1,000 V)	1,812,143	69,095	3.8	38,366	30,729
T5 (1,300 V)	1,709,290	97,417	5.7	60,815	36,602
Average^c^	1,715,197 ± 183,218	75,823 ± 17,768	4.21 ± 0.72	55,894 ± 14,293	19,928 ± 5,880

RNP, ribonucleoprotein.

a Numbers of insertions analyzed.

b Numbers of deletions analyzed.

c Values of average and standard deviation error.

The mutation pattern for each target site was further characterized. The distribution of the five most frequent alleles observed around the cleavage site in *GmCPR5* loci after electro-transfection under different pulsing voltages is presented in **Supplementary Figure 1** (700 V and 1,000 V) and **Figure 4** (1,300 V). gRNAs produced InDels at corresponding target sites, which ranged from +1 to −6 nucleotide (nt) in length, and all induced mutations were observed prevalently at 4th nt upstream of the PAM site except for target sites with few alleles, which were the T1 (1,000 V and 1,300 V), T3 (1,300 V), and T5 (1,000 V) (**Supplementary Figures 1B, C**; **Figures 4B–D**). As shown in **Table 1**, the highest mutation rate was observed only in protoplast samples under 1,300 V, in which the five most frequent alleles were responsible for 6%, 3.14%, and 4% of the total mutation rates for T1 (8.1%), T3 (3.8%), and T5 (5.7%) (**Figures 4B–D**). Further, +1 nt insertion of adenine or thiamine was found prevalent among the observed InDels in the frequent alleles for all the target sites (**Supplementary Figure 1**; **Figure 4**). Apart from this, some of the target sites possessed +1 nt insertion of guanine (T3 under 1,000 V and 1,300 V) and cytosine (T5 under 1,300 V) (**Supplementary Figure 1C**; **Figures 4C, D**). A +2 nt insertion (thiamine and adenine) was also noted for T1 under 700 V (**Supplementary Figure 1A**). A maximum of −5- and −6-bp deletions were observed for T1 (1,000 V) and T5 (1,000 and 1,300 V) following treatments, respectively (**Supplementary Figures 1B, D**; **Figure 4D**). In summary, all the mutant alleles have frameshift mutations, which would result in a complete loss of CPR5 protein function compared to the wild-type alleles of *GmCPR5*. The result analysis from targeted deep sequencing demonstrated that the electro-transfection of RGEN RNPs using the Neon system can be used for site-directed mutagenesis in soybean protoplasts.

## Discussion

The CRISPR/Cas9 technology has emerged as a powerful tool for genome editing and crop improvement. Transient gene expression in leaf mesophyll-derived protoplasts is an excellent resource for genetic manipulation and genome editing using CRISPR/Cas9, which enables the high-throughput analysis of gene functions (Zhu et al., 2016; Xu et al., 2020; Yu et al., 2021). The efficiency of genome editing highly relies on the transfection system and delivery methods including PEG-mediated, electroporation, and microinjection, which have been utilized to introduce DNA, RNA, or protein into plant cells (Masani et al., 2014; Subburaj et al., 2016; Lee et al., 2020; Yeap W. et al., 2021). In addition to electroporation and microinjection, PEG-mediated transient expression technology has been predominantly applied in both model and non-model plants due to their high transfection efficiency as reviewed by Zhang et al. (2021). To date, there have been no reports of CRISPR/Cas9-mediated genome editing via electroporation in soybean species, although several studies have utilized it for transfecting plasmid DNA into soybean protoplasts (Christou et al., 1987; Lin et al., 1987; Dhir et al., 1991). In this study, we standardized the protocols for the delivery of CRISPR/Cas9 RNPs into soybean protoplasts using the Neon electroporation system for the first time. The detection of site-directed mutations in endogenous targeted genes in this work would provide an additional and alternative methodology to the PEG-mediated transient expression technology-based genome editing of the soybean and their related species.

A stable protoplast isolation method and choice of protoplast source with high-quality cells are required for efficient transient gene expression studies (Huang et al., 2013; Li et al., 2018; Lee et al., 2020). Recently, we developed a successful protoplast isolation method for unifoliate leaves of soybean (Subburaj et al., 2022). Following this method, we isolated the protoplasts from trifoliate leaves and obtained a high quantity (2 × 10^6^ cells) and viability of protoplasts (70% ± 2.1%) in this study. While ensuring high transfection efficiency in protoplast cells, and at the same time retaining viability and ability to differentiate (cell division), we have tested the protoplasts of both unifoliate and trifoliate to the different pulsing voltages of 500 V to 1,000 V in a pilot study. It was found that trifoliate cells endure and survive under electrophoretic conditions compared to the unifoliate cells, as they were greatly damaged after electroporation, even at 500 V. In addition, the isolated trifoliate cells had exhibited first protoplast divisions at 5 days after culture initiation as noted for the unifoliate cells in our previous study (Subburaj et al., 2022). Cotyledon and zygotic embryo-derived protoplasts were used in earlier soybean studies for the electroporation-mediated transient expression of introduced DNA molecules (Christou et al., 1987; Lin et al., 1987; Dhir et al., 1991). In this study, we suggest that trifoliate leaf-derived protoplasts of Neon electro-transfection would facilitate DNA-free gene editing using the CRISPR/Cas9 system because of its easy isolation and manipulation.

Electroporation experiments often need an appropriate buffer to provide conductivity as well as cell survival. Therefore, we have investigated the impact of electroporation buffers on protoplasts survival by electroporating them with three different buffers including MMG solution, Neon R, and HEPES buffer as those reported in earlier studies (Dhir et al., 1992; Bhowmik et al., 2018; Lee et al., 2020). Soybean protoplasts did not survive Neon R and HEPES buffers but only survived in MMG solution following electro-transfection, similar to the observed results in cabbage protoplasts (Lee et al., 2020). Upon electroporation with optimized protocol, our results revealed that cell survival and division efficiency decreased with increased pulse voltage. At the same time, the transfection efficiency was increased when the protoplasts were subjected to increasing pulse voltage. This difference might be due to the high electrical pulse applied to protoplast membranes, but more likely, the cellular damage could be induced by increasing voltage, which results in cell death. Many transient pores could also be created on the protoplast membranes under high voltage, which allows the uptake of exogenous GFP-Cas9, thereby increasing the efficiency of transfection. The relative frequency of cell viability was the highest at 500 V (76%) and the lowest at 1,300 V (57%), suggesting that high voltages could decrease the survival rate of cells. Similarly, Bhowmik et al. (2018) also reported that the microspore survival increased with decreasing voltage, with the highest microspore survival of 50% noted at 500 V compared to 1,000 V. Moreover, the efficiency of cell division following 0 V and 500 V was higher than that of other treatments such as 700 V, 1,000 V, and 1,300 V, indicating that the pulsing voltages did not affect the process of mitotic divisions of protoplasts, as they have undergone primary cell divisions at 5 days after culture initiation. In wheat microspores, the transfection efficiency has been noted to increase with decreasing voltage (Bhowmik et al., 2018). In contrast, in cabbage protoplasts, InDel frequencies increased with increasing voltage (Lee et al., 2020). GFP-tagged Cas9 was electro-transfected into soybean protoplasts, the GFP-Cas9 signal was successfully detected in intracellular compartments of electroporated protoplasts, and the highest transfection efficiency was noted under 1,300 V (∼ 40%) compared to other treatments (∼ 20%–23%), indicating that electro-transfection efficiency increased with increasing pulse voltage in soybean protoplasts. This has also corresponded well with the observed InDel frequency rates from targeted deep sequencing results. The InDel frequency of *GmCPR5* following sgRNA (T1, T3, and T5) transfection was increased with increasing pulse voltage from 700 V or 1,000 V to 1,300 V. In this study, the calculated InDel frequencies of T1 (700 V) and T5 (1,000 V) were slightly higher than the observed InDel frequencies of 1.2% (750 V) and 3.4% (1,000 V) for *PDS1* gene in cabbage protoplasts following electro-transfection (Lee et al., 2020). By PEG-mediated transfection of RNPs, a very low editing efficiency has been achieved in protoplasts of several plant species including cabbage (0.09%–2.25%) (Murovec et al., 2018), Cavendish banana (0.19%– 0.92%) (Wu et al., 2020), wild tobacco (0.01–0.9%) (Kim et al., 2017), and grapevine (0.1%) (Malnoy et al., 2016). Further, the targeted deep sequencing results showed that three sgRNAs (T1, T3, and T5) were induced with various mutation pattern sizes ranging from +1 to −6 nt in length at targeted sites, which were found similar to our previous mutagenic CRISPR/Cas9 study in soybean using PEG-mediated delivery method (Subburaj et al., 2022). Taken together, in this study, by electro-transfection, we achieved much higher mutation rates (2.1%–8.1%), suggesting that electro-transfection may potentially be helpful and applicable to the abovementioned plant species to improve editing efficiency compared with the PEG-mediated method.

The high pulse voltage of 1,400 V or 1,250 V has been reported to be lethal to wheat microspores and cabbage protoplasts (Bhowmik et al., 2018; Lee et al., 2020). In this study, we attempted to electro-transfect the soybean protoplasts at 1,300 V and found that trifoliate leaf-derived protoplasts could survive well in post-electroporation, as it had shown 57% viability and ability to divide in culture. However, this study expects further improvements to increase transfection efficiency with a progressive decrease in pulsing voltage within 1,000 V. The regeneration of protoplasts is highly necessary for DNA-free genome editing systems. In this study, after 12 days in culture, dividing protoplasts could not form colonies regardless of treatment of pulsing voltages. This is probably due to the constraining factors including the recalcitrant nature of soybean, genotype-dependent response to culture conditions, type of explant, and age (Wei and Xu, 1988; Cutler et al., 1991; Dhir et al., 1992; Eeckhaut et al., 2013). As demonstrated in previous studies (Wei and Xu, 1988; Dhir et al., 1991), well-established protocols exist for the regeneration of soybean protoplasts. Perhaps a genotype of the perfect donor species or cultivar of soybean is advisable to improve the chances of regeneration of gene-edited protoplasts. In addition, the reduction of non-transfected cells by enriching transfected cells through the application of a fluorescence-activated cell sorting (FACS) system could also have a positive effect on the regeneration of successfully edited cells. Altogether, the protoplast-based gene editing through the Neon electro-transfection system described in this study provides an alternative gene editing platform to the PEG-mediated system for evaluating the efficacy of CRISPR systems as well as gene functional validations in soybean and other related species.

## Conclusion

Our study demonstrated that the CRISPR/Cas9 DNA-free genome editing is effective and efficient in editing soybean genes using the Neon electroporation system. In this study, we demonstrated a time- and cost-efficient *in vitro* electro-transfection assay that provides a rapid assessment and evaluation of gRNA efficiency in soybean protoplasts. The balance between higher voltages and higher targeted mutagenesis will be the challenge for future applications of this method. Nevertheless, a DNA-free transformation system in soybeans to generate non-transgenic gene-edited mutants is highly desired to reduce the occurrence of vector backbone spurious introgressions. This enabling platform for genome editing may accelerate the exploration of gene function for trait improvement in soybean lines. In addition, our study offers new insights into other related species, such as the pinto bean and other *Phaseolus* species, that share similar limitations in genetic transformation and inefficient tissue culture propagation and regeneration processes using other non-transgenic approaches.

## Data availability statement

The datasets presented in this study can be found in online repositories. The names of the repository/repositories and accession number(s) can be found below: NCBI Bioproject accession PRJNA983990.

## Author contributions

SS: Conceptualization, Data curation, Formal Analysis, Investigation, Methodology, Software, Validation, Visualization, Writing – original draft, Writing – review & editing. SA-T: Conceptualization, Formal Analysis, Funding acquisition, Project administration, Resources, Supervision, Writing – review & editing.

## Supplementary material

Supplementary Figure 1 CRSIPR/Cas9-mediated editing of the exogenous *GmCPR5* gene in Soybean protoplasts using electro-transfection and characterization of five most frequent mutation patterns. **(A)** Target sites of T1 at *GmCPR5* loci by electro-transfection at 700 V. **(B)** T1 at *GmCPR5* loci by electro-transfection at 1000 V. **(C)** T3 at *GmCPR5* loci by electro-transfection at 1000 V. **(D)** T5 at *GmCPR5* loci by electro-transfection at 1000 V. Wild type (WT) nuclease target sequences were in bold and underlined. PAM sites denoted by red.
