# Supplementary figures and images for: Establishment of targeted mutagenesis in soybean protoplasts using CRISPR/Cas9 RNP delivery via electro−transfection
Source: Front Plant Sci. 2023 Sep 29;14:1255819. doi: 10.3389/fpls.2023.1255819 (PMC10570537; doi:10.3389/fpls.2023.1255819)

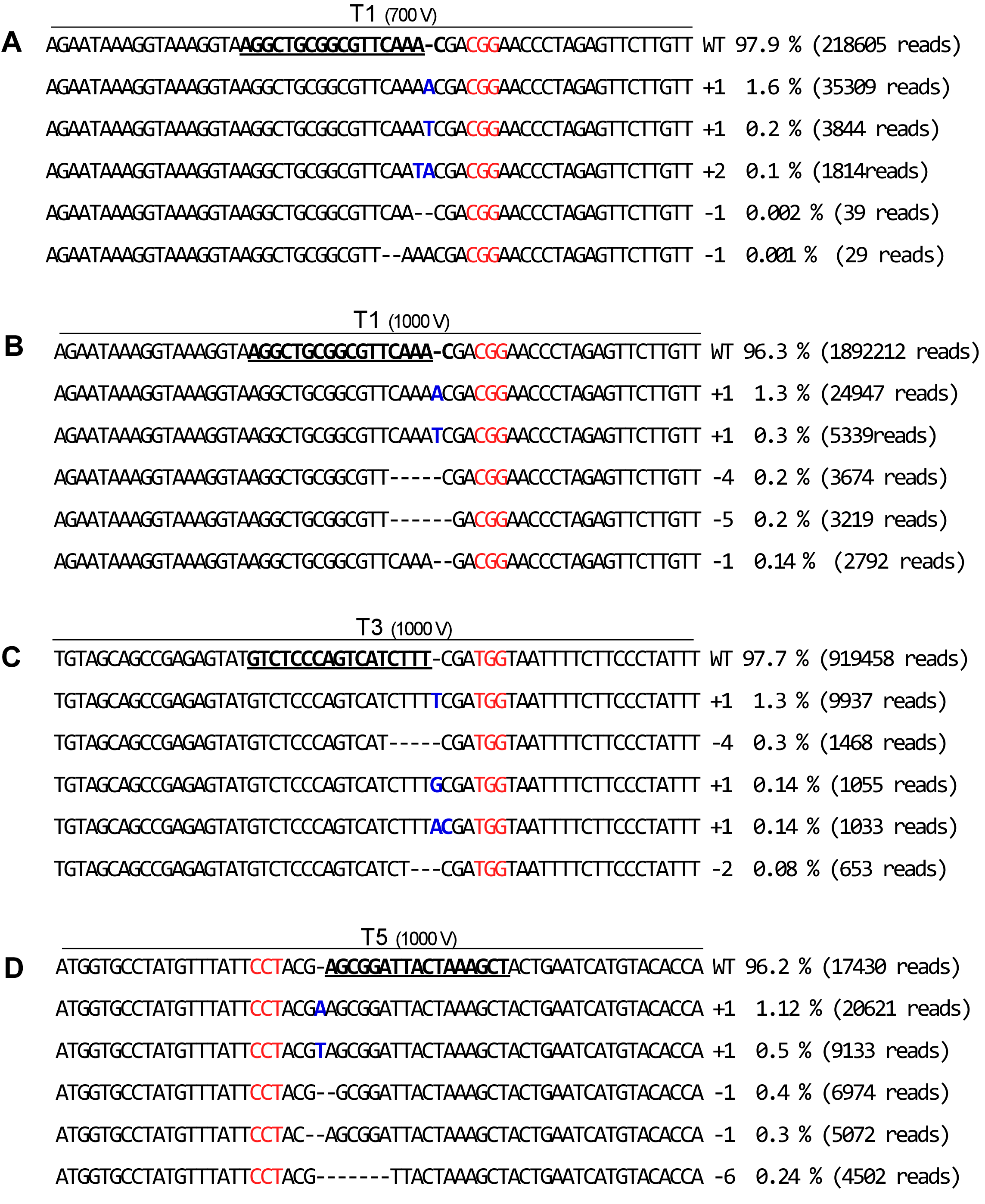

Supplement: Supplementary Figure 1 — CRSIPR/Cas9-mediated editing of the exogenous GmCPR5 gene in Soybean protoplasts using electro-transfection and characterization of five most frequent mutation patterns. (A) Target sites of T1 at GmCPR5 loci by electro-transfection at 700 V. (B) T1 at GmCPR5 loci by electro-transfection at 1000 V. (C) T3 at GmCPR5 loci by electro-transfection at 1000 V. (D) T5 at GmCPR5 loci by electro-transfection at 1000 V. Wild type (WT) nuclease target sequences were in bold and underlined. PAM sites denoted by red. [file Image_1.tif]
