# Supplementary material for: Establishment of targeted mutagenesis in soybean protoplasts using CRISPR/Cas9 RNP delivery via electro−transfection
Source: Front Plant Sci. 2023 Sep 29;14:1255819. doi: 10.3389/fpls.2023.1255819 (PMC10570537; doi:10.3389/fpls.2023.1255819)
Supplement: Supplementary file 2 [file Table_1.docx]

| **Target site** | **T7E1 and Deep Target sequencing (1^st^ PCR)** | | | | **Deep Target sequencing (2^nd^ PCR)** | | | |
| --- | --- | --- | --- | --- | --- | --- | --- | --- |
|  | **Primer Sequence (5'-3')** | **Annealing temperature**  **(Tm °C)** | **Annealing**  **Regions (bp)** | **Amplicon size**  **(bp)** | **Primer Sequence (5'-3')** | **Annealing temperature**  **(Tm °C)** | **Annealing**  **Regions (bp)** | **Amplicon size**  **(bp)** |
| T1 | **F:**CAAATACGGGAACTAGGCGG | 62 | 234-214^a^ | 657 | **F:**CGCAAAGTTCAGAAGCCTCC | 58 | 86–105 | 148 |
|  | **R:**AGCGTTAAAGAAACACGTGA | 61 | 403–423 |  | **R:**GCCAAGAGGAAGGCCAATAG | 56 | 214–234 |  |
| T3 | **F:**CGCAAAGTTCAGAAGCCTCC | 58 | 86–105 | 660 | **F:**TTGCTTGATATTGGGCATTAC | 55 | 508–528 | 237 |
|  | **R:**CCAAACATGCGCTTCATCAC | 60 | 726–745 |  | **R:**CCAAACATGCGCTTCATCAC | 57 | 726–745 |  |
| T5 | **F:**TCTCCTGAAGAGAGCAGGGA^b^ | 57 | 1601–1620 | 693 | **F:**ACGAGGCATGGTGAACTCAA | 57 | 1937–1956 | 148 |
|  | **R1:**AACTGCCAATTGAGCAAAGG^b^ | 56 | 2275–2294 |  | **R:**TGCCTGGGTTGATGGTGTAC | 58 | 2067–2085 |  |

**Supplementary Table 1**. List of PCR primers used in this study
